# Supplementary material for: Timosaponin A3 Inhibits Palmitate and Stearate through Suppression of SREBP-1 in Pancreatic Cancer
Source: Pharmaceutics. 2022 Apr 27;14(5):945. doi: 10.3390/pharmaceutics14050945 (PMC9147344; doi:10.3390/pharmaceutics14050945)
Supplement: Supplementary file 1 [file pharmaceutics-14-00945-s001.zip › pharmaceutics-1564084-supplementary.pdf]

pharmaceutics

Supplementary Materials:
Inhibition of SREBP-1 Transcriptional Activity by Timosaponin A3 Induces Fatty Acid Biosynthesis Deficiency and Growth Inhibition in Pancreatic cancer

Yumi Kim[1,2], Eun-Jin An[1,2], Hyun Min Ko[1,2], Ji hoon Jung[1,2], Yun-Cheol Na[3], and Hyeung-Jin Jang[1,2,*]

[1] College of Korean Medicine, Kyung Hee University, 26 Kyungheedae-ro, Dongdaemun-gu, Seoul, 02447, Republic of Korea
[2] Department of Science in Korean Medicine, Graduate School, Kyung Hee University, 26 Kyungheedae-ro, Dongdaemun-gu, Seoul, 02447, Republic of Korea
[3] Western Seoul Center, Korea Basic Science Institute, 150 Bugahyeon-ro, Seodaemun-gu, Seoul, 03759, Republic of Korea

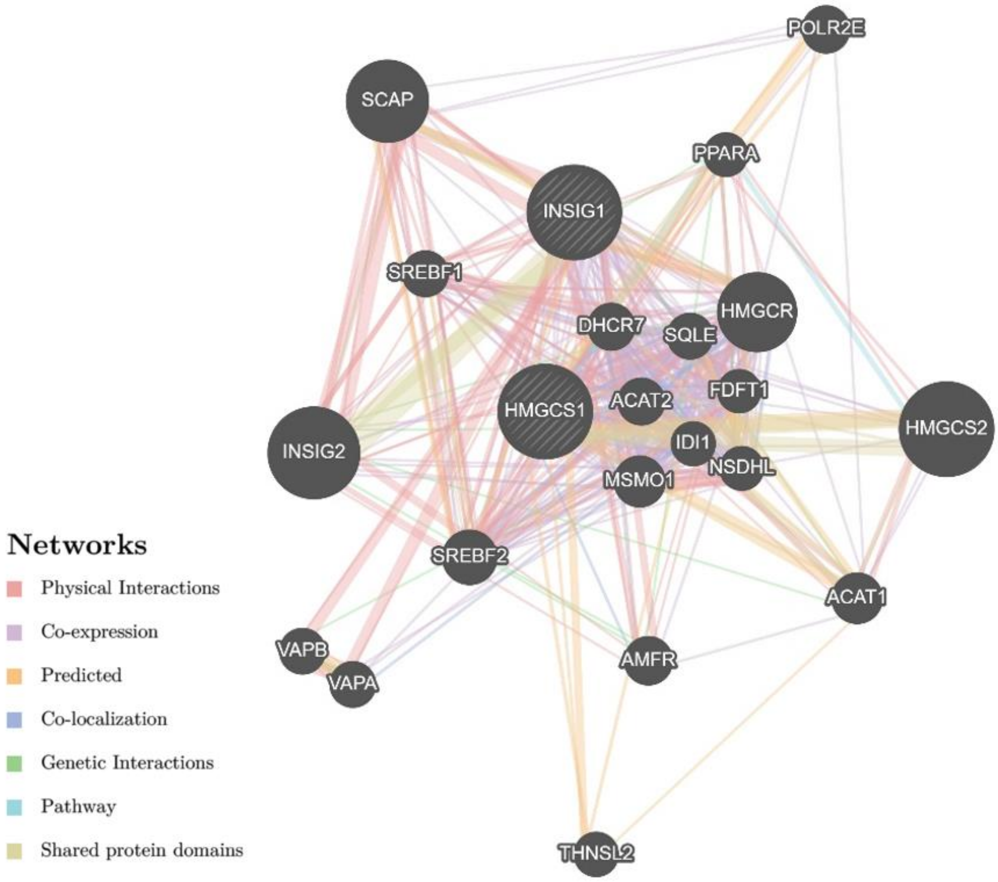

Figure S1. Gene network produced using GeneMANIA. The network consists of INSIG-1 and HMGCS-1 genes in human were constructed by GeneMANIA online source. The 20 interaction genes between INSIG-1 and HMGCS-1 were surveyed in term of physical interaction, co-expression, predicted, co-localization, genetic interactions, genetic interactions, pathway, and shared protein domains. The thickness of the colored line of each network interaction indicates the interaction strength.

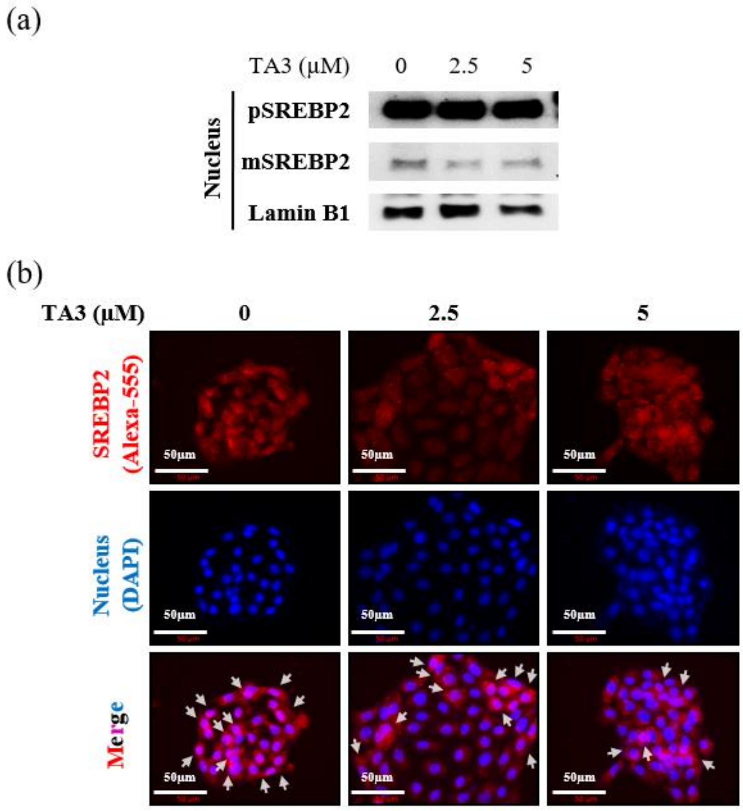

Figure S2. Effect of TA3 on the expression of precursor and mature SREBP-2 in BxPC-3 cells. (**a**) The BxPC-3 cells were treated with TA3 (2.5 and 5 μM) for 12 h. Nuclear extracts were analyzed by immunoblotting. (**b**) After TA3 treatment for 12 h, an immunofluorescence assay was performed to analyze the effect on SREBP-2 nuclear translocation in BxPC-3 cells. Arrows denote nuclear translocation. The magnification is 400×. Scale bar, 50μm.

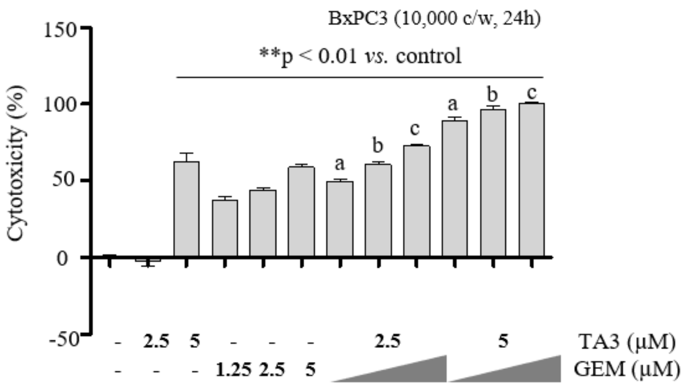

Figure S3. Cytotoxicity effect of combination treatment with TA3 and Gemcitabine (GEM) measured by MTT assay. The BxPC3 cells were treated TA3 and GEM separately or together for 24 h. Cytotoxicity presented as percentage with that of the negative control, with the mean ± SD of three independent experiments (a: $p < 0.01$, *vs*. 12.5 μM GEM; b: $p < 0.01$ *vs.* 2.5 μM GEM; c: $p < 0.01$ *vs.* 5 μM GEM).

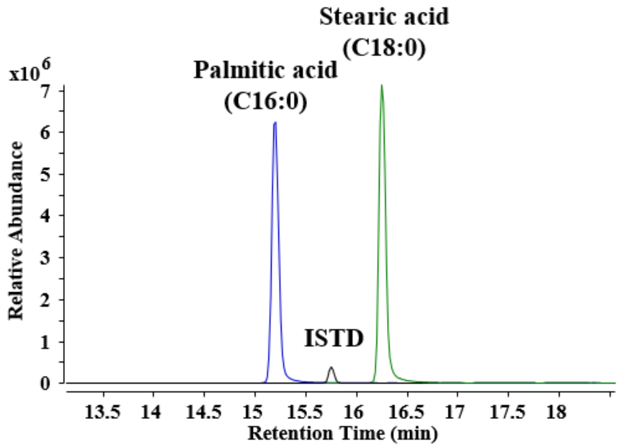

Figure S4. Total ion chromatograms of palmitic acid and stearic acid in standard mixtures (10 μg/ml) with internal standard (1 μg/ml).

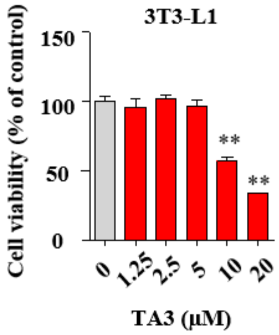

Figure S5. Effect of TA3 on 3T3-L1 cell viability measured by MTT assay. The cells were treated with TA3 at the various concentration for 24 h. Data are shown as the mean ± SD (**$p < 0.01$, *vs.* Control).
